# Impact of interdisciplinary tumor boards (ITB) and personalized treatment on survival outcomes in metastatic castration-resistant prostate cancer

**DOI:** 10.1007/s00432-025-06135-8

**Published:** 2025-03-06

**Authors:** Laura Lawaczeck, Anna Rüdiger, Jörg Hennenlotter, Joël Hammes, Valentina Spingler, Simon Walz, Eva Erne, Igor Tsaur, Steffen Rausch

**Affiliations:** Department of Urology, Klinik Für Urologie, Eberhard-Karls-University, Universitätsklinik Tübingen, Hoppe-Seyler-Straße 3, 72076 Tübingen, Germany

**Keywords:** Prostate cancer, mCRPC, Interdisciplinary tumor board, ITB, Personalized treatment, Individual treatment

## Abstract

**Purpose:**

Interdisciplinary tumor boards (ITB) are essential in optimizing treatment recommendations for metastatic castration-resistant prostate cancer (mCRPC) by incorporating oncology guidelines, clinical trials, and patient-specific factors to ensure individualized care. This study examines clinical parameters that influence ITB recommendations, evaluates their adherence to guidelines, and assesses their impact on patient survival.

**Methods:**

In a retrospective analysis, data from 187 mCRPC patients discussed at an ITB in a tertiary care center in 2018 were evaluated. Patient- and disease-specific factors were correlated with adherence to National Comprehensive Cancer Network^®^ (NCCN^®^) guidelines and overall survival (OS). The impact of clinical parameters on survival outcomes was assessed through univariate and multivariate analyses.

**Results:**

The median patient age was 72.8 years, with a median prostate-specific antigen (PSA) level of 65.0 ng/ml. Guideline-compliant recommendations were given in 42.9% of cases, while 57.1% received individualized recommendations. Clinical trial eligibility was noted in 24.8% of patients. Individualized ITB recommendations were associated with significantly longer OS (38.3 vs. 21.2 months, p = 0.03). Shorter OS correlated with renal impairment (p = 0.007), symptomatic metastases (p < 0.0001), and visceral metastases (p < 0.0001). Limitations include the retrospective design, lack of follow-up on therapy adherence, and absence of progression-free survival (PFS) data.

**Conclusion:**

ITB discussions improve survival in mCRPC patients, mainly due to personalized approaches and better access to clinical trials. Visceral and symptomatic metastases as well as renal impairment are risk factors for reduced OS, emphasizing the need for careful management of these high-risk patients. The results support the expanded use of ITB to improve mCRPC treatment outcomes.

**Supplementary Information:**

The online version contains supplementary material available at 10.1007/s00432-025-06135-8.

## Introduction

Metastatic castration-resistant prostate cancer (mCRPC) is characterized by disease progression despite ongoing androgen deprivation therapy (ADT) (Morote et al. 2022; Saad and Hotte 2010; Guidelines 2023). Over recent years, numerous treatment options have emerged for mCRPC, including next-generation hormonal agents such as abiraterone acetate (Procopio et al. 2022) and enzalutamide (Tagawa et al. 2021), poly (ADP-ribose) polymerase (PARP) inhibitors (Mateo et al. 2020; Bono et al. 2020), prostate-specific membrane antigen (PSMA)-targeted therapies (Sartor et al. 2021), radionuclide therapies with radium-223 (Nilsson 2016) and taxane-based chemotherapies (Paller and Antonarakis 2011).

To ensure optimal treatment strategies for mCRPC patients, interdisciplinary tumor boards (ITB) at uro-oncologic centers involve specialists from various disciplines, including urology, oncology, radiation oncology, pathology, radiology, and nuclear medicine. These multidisciplinary discussions, grounded in evidence-based clinical data, lead to personalized treatment recommendations for each patient (Heidenreich 2019). Furthermore, ITB consider clinical trial opportunities as part of the decision-making process, with established guidelines serving as the foundation of their recommendations (Saghir 2014).

When multiple treatment options are available, ITB integrate patient-specific and disease-specific variables to guide their decisions. While not all clinical factors are based on high-level evidence from clinical trials, the collaborative approach of an ITB offers the best framework for managing complex cancer cases (Specchia et al. 2020). Despite the increasing reliance on ITB, there is still limited evidence regarding their direct impact on clinical outcomes, particularly in terms of survival and quality of life (Specchia et al. 2020). Nonetheless, discussions in ITB are often associated with enhanced patient satisfaction and improved clinical results (Saghir 2014; Huang et al. 2024).

This study aims to assess the impact of ITB recommendations on clinical outcomes in patients with castration-resistant prostate cancer (CRPC). We also examine the extent to which these recommendations align with established clinical guidelines, integrating patient-specific and disease-specific factors.

## Material and methods

**Fig. 1:**
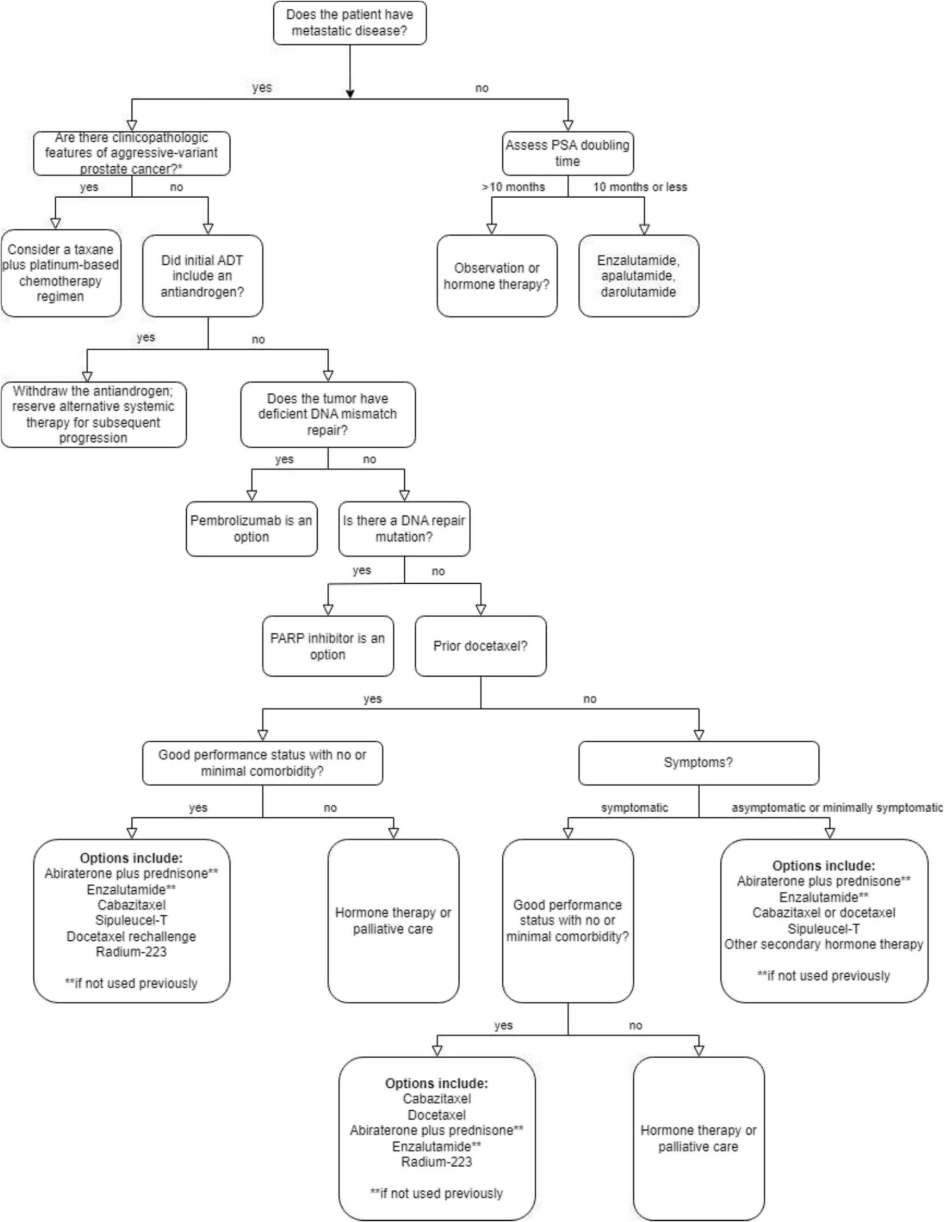
Treatment algorithm of metastatic castration-resistant prostate cancer modified according to National Comprehensive Cancer Network (NCCN^®^) (Mohler, et al. 2019; NCCN^®^, NCCN^®^ Guidelines Version 2022). *Aggressive-variants of prostate cancer, according to the NCCN^®^ guidelines version 3.2022, include small cell and neuroendocrine prostate cancer. *PSA* prostate-specific antigen, *ADT* androgen deprivation therapy, *DNA* deoxyribonucleic acid, *PARP* poly (ADP-ribose) polymerase

A total of 187 consecutive patients with CRPC were included in the study after being discussed at an ITB within a tertiary uro-oncologic reference center between January 2018 and December 2019. The ITB takes place weekly and involve a multidisciplinary team comprising urologists, radiation oncologists, radiologists, pathologists, internists, and nuclear medicine specialists. Patient-specific and disease-specific parameters—such as age, histology, metastasis patterns, symptoms, PSA kinetics, the number and type of previous therapies, renal function (assessed using the glomerular filtration rate (GFR) > 60 ml/min based on the MDRD formula), and hemoglobin levels (> 10.8 g/dl)—were collected. ITB recommendations and their conformity with contemporary guidelines (NCCN® guidelines version 3.2022) (see Fig. 1) were evaluated (Mohler, et al. 2019; NCCN®, NCCN® Guidelines Version 2022). Treatment recommendations were classified as “individual” if they deviated from guideline recommendations or if clinical trial participation was proposed. When more than one individual treatment option was suggested, this was noted accordingly.

Both univariate (Kaplan–Meier analysis, Cox regression model) and multivariate (Cox regression model) analyses were performed to investigate the correlation of patient-specific and disease-specific clinical parameters with overall survival (OS) based on ITB recommendations. Chi-square tests were used to assess associations between categorized patient-specific variables and ITB recommendations. Statistical significance was set at p < 0.05. Statistical analyses were conducted using JMP^®^ 16.2.0 software. The study was approved by the Ethical Committee of the University of Tübingen (922/2020BO2).

## Results

The median age at the time of ITB discussion was 72.8 years (± 0.6 standard error (SE)), and the median PSA level was 65.0 ng/ml (± 46.8 SE) (see Table 1). In total, 76.1% of patients had a Gleason score ≥ 8, and 73.5% exhibited a PSA doubling time (DT) of less than 10 months (see Table 2). Overall, mCRPC status was diagnosed in 96.8% of patients, while 3.2% were diagnosed with non-metastatic CRPC (nmCRPC). In terms of metastatic sites, 87.1% of patients had bone metastases, 26.0% had visceral metastases, and 64.7% presented with lymph node involvement. At initial diagnosis, synchronous metastases were observed in 40.6% of cases, and 67.1% of the patients exhibited symptomatic metastases at the time of the ITB discussion. Further details on metastatic patterns are provided in Table 2. Additionally, 42.5% of patients received osteoprotective therapy, either bisphosphonates or denosumab.

**Table 1:** Patient collective and clinical characteristics (*n* = 187)

Median/mean age in years (range)	72.8/72.4	(49.2–89.3)
Median/mean PSA-value in ng/ml (range)	65.0/266.3	(0.09–4899.0)
Median/mean hemoglobin-value in g/dl (range)	10.8/10.8	(5.2–16.5)
Median/mean creatinine-value in mg/dl (range)	0.8/1.1	(0.4–6.0)

*PSA* prostate-specific antigen

**Table 2:** Patient-specific and disease-specific variables

Patient-specific and disease-specific variables	Yes in %/(*n*)	No in %/(*n*)
Gleason-score ≥ 8	76.1% (121)	23.9% (38)
Renal impairment (GFR < 60 ml/min)	20.6% (26)	79.4% (100)
Synchronous metastases	40.6% (73)	59.4% (107)
PSA doubling time < 10 months	73.5% (83)	26.5% (30)
Bone metastases	87.1% (149)	12.9% (22)
Visceral metastases	26.0% (38)	74.0% (108)
Lymph node metastases	64.7% (121)	35.3% (66)
Symptomatic metastases	67.1% (102)	32.9% (50)
Guideline-compliant recommendation	42.9% (76)	57.1% (101)
Clinical trial option	22.6% (40)	77.4% (137)
ECOG performance status 0 or 1	68.8% (22)	31.2%/(10)
ECOG performance status ≥ 2	31.2%/(10)	68.8% (22)

*GFR* glomerular filtration rate, *PSA* prostate-specific antigen, *ECOG* Eastern Cooperative Oncology Group, *n* number, % percent

At the time of the ITB discussion, all patients were receiving ADT. A total of 13.9% had previously undergone docetaxel chemotherapy in the hormone-sensitive setting (see Table 3). A total of 41.2% of the patients were in the first-line mCRPC treatment setting, whereas 58.8% had undergone prior treatment and were being referred for recommendations on subsequent therapies. A summary of prior treatments before the ITB discussion is provided in Table 3. Notably, 16.6% of the cohort had received radium-223 dichloride, and 7.0% had undergone PSMA-ligand therapy.

**Table 3:** Prior treatment sequence at ITB recommendation and treatment recommendation from ITB

Prior treatment at ITB recommendation (n = 187)	*n*	%
**Prior treatment at ITB recommendation in mHSPC**	***77***	***41.2***
Androgen deprivation therapy (ADT) only	51	27.3
ADT + upfront-chemotherapy (taxane-based)	26	13.9
**Prior treatment at ITB recommendation in mCRPC**	***110***	***58.8***
ADT + chemotherapy (taxane-based)	7	3.7
ADT + abiraterone	16	8.6
ADT + enzalutamide	6	3.2
ADT + abiraterone/enzalutamide (or reverse)	12	6.4
ADT + abiraterone/chemotherapy (taxane-based) (or reverse)	10	5.4
ADT + enzalutamide/chemotherapy (taxane-based) (or reverse)	15	8.0
ADT + abiraterone/enzalutamide/chemotherapy (taxane-based) (or reverse)	33	17.6
Other treatment (radiotherapy, clinical trial, platin-based chemotherapy)	11	5.9
Additional Radium-223 dichloride treatment	31	16.6
Additional PSMA-ligand therapy	13	7.0

Individual treatment recommendation from ITB (*n* = 101)	*n*	%
PSMA-ligand therapy	42	41.6
Novel hormonal therapy (NHT)	38	37.6
Clinical trial participation	25	24.8
Metastasis-directed radiotherapy	15	14.9
Radium-223 dichloride treatment	5	5.0
PARP-inhibitor therapy	3	3.0

*ADT* Androgen deprivation therapy, *mHSPC* metastatic hormone-sensitive prostate cancer, *mCRPC* metastatic castration-resistant prostate cancer, *PSMA* = prostate-specific membrane antigen, *NHT* novel hormonal therapy, *ITB* interdisciplinary tumor boards, *PARP* poly (ADP-ribose) polymerase), *n* number, % percent

In ITB discussion a guideline-compliant recommendation was made in 42.9% of cases, while 57.1% received individualized treatment recommendations (see Table 2). Among the individualized therapies, PSMA-ligand therapy was recommended in 41.6% of patients, novel hormonal therapy (NHT) in 37.6%, participation in clinical trials in 24.8%, metastasis-directed radiotherapy in 14.9%, and radium-223 dichloride in 5.0% (see Table 3). PSMA-positron emission tomography/computed tomography (PET/CT), with the potential option of subsequent PSMA-ligand therapy, was only recommended for patients with advanced stages of mCRPC who had undergone chemotherapy with at least docetaxel or a combination of docetaxel and cabazitaxel, or for patients deemed unsuitable for chemotherapy. In our study, PSMA-PET/CT was not recommended as a diagnostic approach for patients with nmCRPC; however, this group comprised only a very small number of patients, and all patients had already undergone imaging according to the NCCN^®^ guidelines before being presented at the ITB for discussion of further treatment options.

**Fig. 2:**
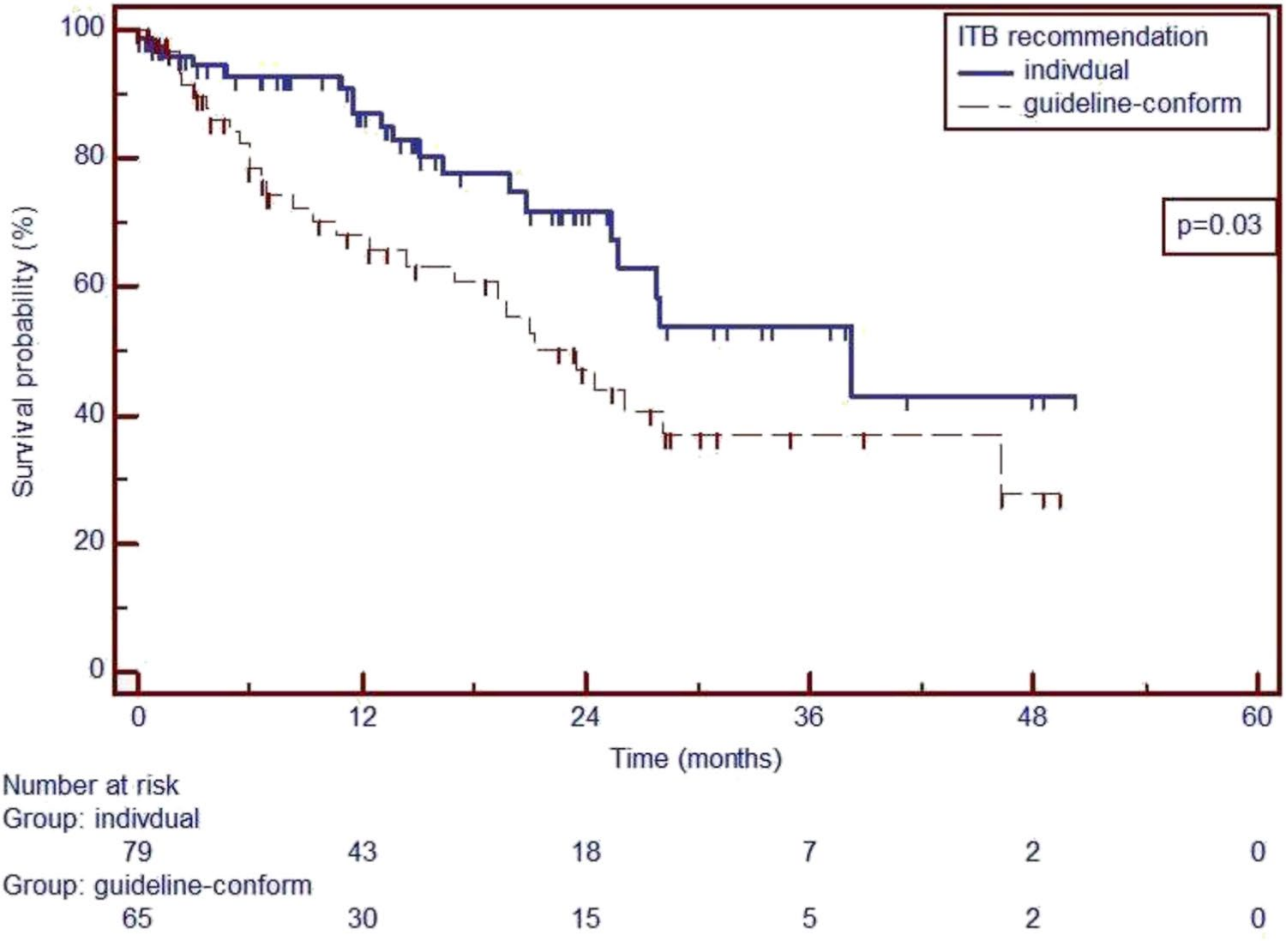
Kaplan–Meier analysis of overall survival (OS) for individual treatment and guideline-compliant treatment. % percent, *p* p-value, *HR* hazard ratio, 95% *CI* 95% confidence interval

The median follow-up after the ITB discussion was 11.8 months (95% confidence interval (CI) 9.9–13.7), while the median follow-up from the initial prostate cancer diagnosis was 94.4 months (95% CI 85.3–103.5). The median time from the initial diagnosis of prostate cancer to the ITB discussion was 82.6 months (95% CI 73.8–91.4). In all mCRPC patients, the median OS was 26.1 months (95% CI 21.2–46.3). Patients receiving individualized ITB recommendations experienced significantly longer OS compared to those receiving guideline-compliant recommendations (38.3 months (95% CI 25.3–not available) vs. 21.2 months (95% CI 12.4–46.3); p = 0.03) (see Fig. 2). Univariate Cox regression analysis identified reduced OS as being associated with renal impairment (GFR < 60 ml/min, p = 0.007), symptomatic metastases (p < 0.0001), and visceral metastases (p < 0.0001) (see Table 4). In multivariate Cox regression analysis, symptomatic (p = 0.006) and visceral metastases (p = 0.001) remained independent predictors of reduced OS (see Table 4). No significant correlation was found between patient- and disease-specific clinical variables and the choice of ITB recommendation (all p > 0.05) (see Supplementary Table 5).

**Table 4:** Correlation of overall survival with clinical variables from univariate and multivariate Cox regression analyses

Cox regression analyses	univariate			multivariate		
	HR	95% CI	p-value	HR	95% CI	p-value
Individual treatment	0.53	0.29–0.94	**0.030**	0.79	0.35–1.79	0.580
Renal impairment (GFR < 60 ml/min)	2.69	1.39–5.21	**0.007**	2.31	1.00–0.188	0.062
Symptomatic metastases	4.37	2.19–8.72	** < 0.0001**	3.37	1.35–10.32	**0.006**
Visceral metastases	4.44	2.39–8.23	** < 0.0001**	4.65	1.94–11.11	**0.001**
Gleason-Score ≥ 8	0.81	0.41–1.61	0.571			
Synchronous metastases	0.97	0.54–1.75	0.926			
Metachronous metastases	2.41	0.33–17.47	0.314			
PSA doubling time < 10 months	1.04	0.51–2.13	0.904			
Available clinical trial option	1.03	0.54–1.98	0.924			
Age ≥ 72.7 years	1.24	0.71–2.17	0.450			

*PSA* prostate-specific antigen, *GFR* glomerular filtration rate, *HR* hazard ratio, 95% *CI* 95% confidence interval

The statistically significant values are highlighted in bold

## Discussion

The results of this study demonstrate the value of interdisciplinary tumor board (ITB) discussions for patients with mCRPC. In this cohort, 42.9% of ITB recommendations adhered to established guidelines, while 57.1% deviated towards individualized treatment decisions. The fact that a substantial proportion of patients received non-standard recommendations highlights the importance of multidisciplinary input in ITB, which allows for patient-specific tailoring of treatment regimens. Interestingly, patients who received individualized treatment recommendations exhibited significantly longer OS, underscoring the clinical benefit of such approaches.

Several mechanisms may explain how ITB discussions improve patient outcomes. These include access to a broader range of expert opinions, a reduced risk of human error, and more coordinated care resulting from the involvement of multiple specialists (Huang et al. 2024). Furthermore, many novel or not yet fully approved therapies may be offered to patients via ITB consultations. A key example in our study is the early indication for PSMA-ligand therapy, which was recommended in 41.6% of patients, significantly prior to its official approval (Sartor et al. 2021; Keam 2022; Sar et al. 2023). During the study period, PSMA-ligand therapy had not yet received approval from the European Medicines Agency (EMA), which was granted only in December 2022 (Heilinger, et al. 2023). Consequently, even for patients who had already undergone chemotherapy with docetaxel and/or cabazitaxel, its use was considered off-label and required a tumor board decision for cost coverage. Noteworthy, the nowadays guideline-compliant decision to perform PSMA-PET/CT and PSMA-ligand therapy in our study was based on tumor board recommendations only and did not reflect NCCN^®^ guidelines at the time. Similarly, olaparib, a PARP inhibitor, was not approved as a monotherapy for mCRPC following a positive Breast Cancer Gene 1 and Breast Cancer Gene 2 (BRCA1/2) mutation analysis, necessitating tumor board recommendations to facilitate cost coverage. In these cases, genomic testing to determine BRCA1/2 mutation status was specifically advised. This highlights the potential of ITB to facilitate access to cutting-edge therapies that might not be available outside of multidisciplinary settings.

In our analysis, 24.8% of patients were offered the option of participating in clinical trials. Clinical trial participation has been shown to improve outcomes for patients with mCRPC, and is strongly advocated by international consensus conferences, such as the Advanced Prostate Cancer Consensus Conference (APCCC) (Gillessen et al. 2023). In addition to clinical trial enrollment, ITB discussions provide access to modern targeted therapies, next-generation imaging, and advanced molecular analyses, especially in late-stage disease (Unger et al. 2016). While clinical guidelines are crucial in ITB decision-making, they are often challenged by the need to integrate novel, high-level evidence that may not yet be fully reflected in current recommendations (Winn et al. 1996).

Our study also identified key predictors of reduced OS in mCRPC, including renal impairment (GFR < 60 ml/min), symptomatic metastases, and visceral metastases. These findings are consistent with prior research demonstrating that visceral metastases, especially in sites such as the liver and lungs, are associated with poor prognosis in prostate cancer. Studies by Budnik et al*.* and Tappero et al*.* similarly reported that patients with visceral metastases had significantly worse survival outcomes compared to those with metastases confined to the bone (Budnik et al. 2019; Tappero 2023). Additionally, our findings align with other research suggesting that patients with multiple visceral metastases experience even poorer outcomes than those with isolated metastasis sites (Cui et al. 2020).

The role of symptomatic metastases as a marker of disease progression is well documented. Symptom onset or worsening in mCRPC is strongly associated with disease progression and shorter survival (Saad et al. 2018). The management of symptomatic metastases often requires individualized care, including interventions such as metastasis-directed radiotherapy (Boyer et al. 2014). In our investigation, 67.1% of patients had symptomatic metastases, and 14.9% received palliative radiotherapy.

Chronic renal impairment is highly prevalent in patients with mCRPC and is associated with increased cancer-related mortality (Launay-Vacher et al. 2016). The impact of renal function on drug pharmacokinetics and treatment toxicity must be carefully considered in treatment planning for this population (Launay-Vacher et al. 2016; Bednarek 2020). This is particularly relevant for agents such as novel hormonal therapies and chemotherapy, which can have detrimental effects on renal function (Bednarek 2020). The complex interplay between drug-drug interactions and renal impairment underscores the need for individualized dosing and therapeutic strategies, often achieved through ITB consultation (Papotti et al. 2021; Leeuwen et al. 2015).

Furthermore, molecular tumor boards (MTB) are gaining importance in mCRPC, particularly as more targeted therapies become available (Slootbeek et al. 2022). Recent studies have shown that genetically matched therapies recommended by MTB can lead to durable responses in a significant proportion of patients (Slootbeek et al. 2022). As biomarkers such as circulating tumor cells (CTC), DNA repair gene alterations, and androgen receptor splice variants become more widely implemented in clinical practice, ITB and MTB discussions will play a pivotal role in guiding personalized treatment for mCRPC (Slootbeek et al. 2022; Asif and Teply 2021).

Despite the strengths of this study, certain limitations must be acknowledged. First, all ITB recommendations were evaluated based on NCCN^®^ guidelines, and any deviations were classified as individual therapies. However, some patients receiving individual therapies may have participated in clinical trials where guideline-compliant therapies were administered as part of control groups. Additionally, data on progression-free survival (PFS) were not available, limiting the ability to assess the impact of ITB recommendations on this important clinical endpoint. Our study is further limited by the fact that ITB registrations from external centers without patient admissions make it impossible to conduct an onsite assessment of the registered patients’ performance status based on the ECOG score in these cases.

Overall, in our study, individual treatment recommendations in ITB lead to improved OS in mCRPC. Therefore, urologists and oncologists treating CRPC patients in their daily practice should be encouraged to forward case discussions in an ITB. To date, utilization of ITB in genito-urinary cancer does not appear to be widespread everywhere (Heidenreich 2019; Atwell et al. 2019). Atwell et al*.* observed heterogeneous ITB referral rates depending on tumor entities at their institution (Atwell et al. 2019). While 90–100% of patients with lung cancer or upper gastrointestinal cancer were referred to ITB, only 34% and 28% of patients with prostate cancer and bladder cancer, respectively, were discussed in an ITB (Atwell et al. 2019).

It is important to note that establishing a formal ITB at regular intervals demands additional work time, as well as administrative and financial resources (Heidenreich 2019). Despite the substantial resource requirements, implementing ITB in specialized uro-oncology centers is beneficial, and both internal and external referrals should be encouraged, especially in the mCRPC scenario (Huang et al. 2024).

## Conclusions

mCRPC is a heterogeneous disease state that requires interdisciplinary management and personalized treatment strategies. Our findings demonstrate that individualized recommendations from ITB discussions are associated with improved overall survival compared to guideline-compliant treatments. In particular, ITB provide crucial access to novel therapies and clinical trials, which may not be accessible in standard clinical practice. Additionally, renal impairment, symptomatic metastases, and visceral metastases were identified as key predictors of reduced survival, highlighting the need for tailored therapeutic approaches in these high-risk populations.

Given the significant improvements in patient outcomes observed in this study, we suggest that urologists and oncologists consider incorporating ITB discussions into routine clinical care for patients with mCRPC. Despite the logistical and financial resources required to conduct regular ITB meetings, the benefits in terms of enhanced survival and access to novel treatments may justify their implementation in dedicated uro-oncologic centers. However, our findings should be confirmed in prospective studies to further validate the positive impact of ITB discussions. Increasing the use of ITB, particularly in genito-urinary cancer, may help optimize outcomes for patients with advanced prostate cancer.

## Supplementary Information

Below is the link to the electronic supplementary material.

Supplementary file1 (DOCX 13 KB)

## Data Availability

The data analyzed in this study are not publicly available due to patient confidentiality and institutional restrictions but can be made available upon reasonable request. Requests for access to the datasets should be directed to the corresponding author, Prof. Dr. med. Steffen Rausch.
