# Supplementary material for: Impact of interdisciplinary tumor boards (ITB) and personalized treatment on survival outcomes in metastatic castration-resistant prostate cancer
Source: J Cancer Res Clin Oncol. 2025 Mar 6;151(3):101. doi: 10.1007/s00432-025-06135-8 (PMC11885382; doi:10.1007/s00432-025-06135-8)
Supplement: Supplementary file 1 — Supplementary file1 (DOCX 13 KB) [file 432_2025_6135_MOESM1_ESM.docx]

**Supplementary Table 5: Correlation of clinical characteristics with guideline-compliant and individual treatment groups**

PSA = prostate-specific antigen, GFR = glomerular filtration rate

|  | **Guideline-compliant treatment (42.9%)** | **Individual treatment (57.1%)** | **p-value** |
| --- | --- | --- | --- |
|  | **n=** | **n=** |  |
| Age ≥72.7 years (yes/no) | 41/35 | 48/53 | 0.39 |
| Gleason-Score ≥8 (yes/no) | 46/21 | 72/16 | 0.06 |
| PSA doubling time <10 months (yes/no) | 40/12 | 43/18 | 0.44 |
| Synchronous metastases (yes/no) | 32/44 | 39/59 | 0.76 |
| Metachronous metastases (yes/no) | 71/5 | 96/3 | 0.28 |
| Bone metastases (yes/no) | 63/8 | 82/14 | 0.53 |
| Visceral metastases (yes/no) | 21/42 | 17/66 | 0.10 |
| Symptomatic metastases (yes/no) | 46/19 | 52/31 | 0.30 |
| Renal impairment (GFR <60 ml/min) (yes/no) | 46/14 | 54/12 | 0.48 |
| Hemoglobin ≥10.8 g/dl (yes/no) | 35/25 | 29/34 | 0.17 |
| Available clinical trial option (yes/no) | 16/60 | 25/76 | 0.67 |
